# Enhancing CRISPR‐Cas12a base editing in plants with LbCas12a variants and introns

**DOI:** 10.1111/jipb.70249

**Published:** 2026-04-02

**Authors:** Yanhao Cheng, Gen Li, Man Zhou, Rushil Mandlik, Doris Wang, Yiping Qi

**Affiliations:** ^1^ Department of Plant Science and Landscape Architecture University of Maryland College Park 20742 MD USA; ^2^ Institute for Bioscience and Biotechnology Research University of Maryland Rockville 20850 MD USA

**Keywords:** adenine base editing, cytosine base editing, improved Cas12a base editors, poplar, rice

## Abstract

Cytosine base editors (CBEs) and adenine base editors (ABEs) are powerful tools for precise genome editing in plants. Conventionally, such base editors are built upon the CRISPR‐Cas9 systems where Cas9 nickases are used. To expand the base editing scope and minimize off‐target effects, base editors derived from the CRISPR‐Cas12a systems are desired. However, the use of deactivated Cas12a (dCas12a) in such base editors constrains the editing activity, preventing the wide use of Cas12a base editors for plant research and trait development. In this study, we demonstrate the use of an ABE based on the efficient LbCas12a‐RRV variant to introduce herbicide‐resistant mutations in *OsACCase* in rice. To improve Cas12a CBEs and ABEs, we inserted introns into the coding sequence of dLbCas12a‐RRV. This intron‐containing Cas12a‐CBE shows substantial improvement in editing efficiency in rice, compared to the intron‐less counterparts. By contrast, the improvement of ABE with the intron‐containing dLbCas12a‐RRV is very limited, partly due to the already high baseline editing efficiency of the intron‐less dLbCas12a‐RRV ABE. Testing of these base editors in poplar shows elevated C‐to‐T base editing by dLbCas12a‐RRV–intron‐CBE. For A‐to‐G editing, ABEs built upon dLbCas12a‐RV and dLbCas12a‐RRV variants showed significant improvement over ABEs derived from wild‐type LbCas12a and the ttLbCas12a variant. The addition of introns to dLbCas12a‐RRV does not further improve the base editing efficiency. With whole genome sequencing in rice, we evaluated genome editing specificities with these improved Cas12a base editors. Our analyses show that both intron‐containing Cas12a CBE and ABE barely introduce guide RNA‐dependent off‐target mutations. However, they can generate guide RNA‐independent off‐target mutations, which are likely attributed to the high enzymatic activities of the deaminases. Collectively, our study demonstrates the successful use of a Cas12a base editor for trait development and reports improved Cas12a CBEs and ABEs for precise base editing in plants.

## Graphical abstract

Intron optimization of LbCas12a‐RRV improves cytosine and adenine base editing efficiency in plants, supports multiplexed and double‐strand break‐free genome modification, and expands precise genome engineering tools for crop breeding and plant functional research.

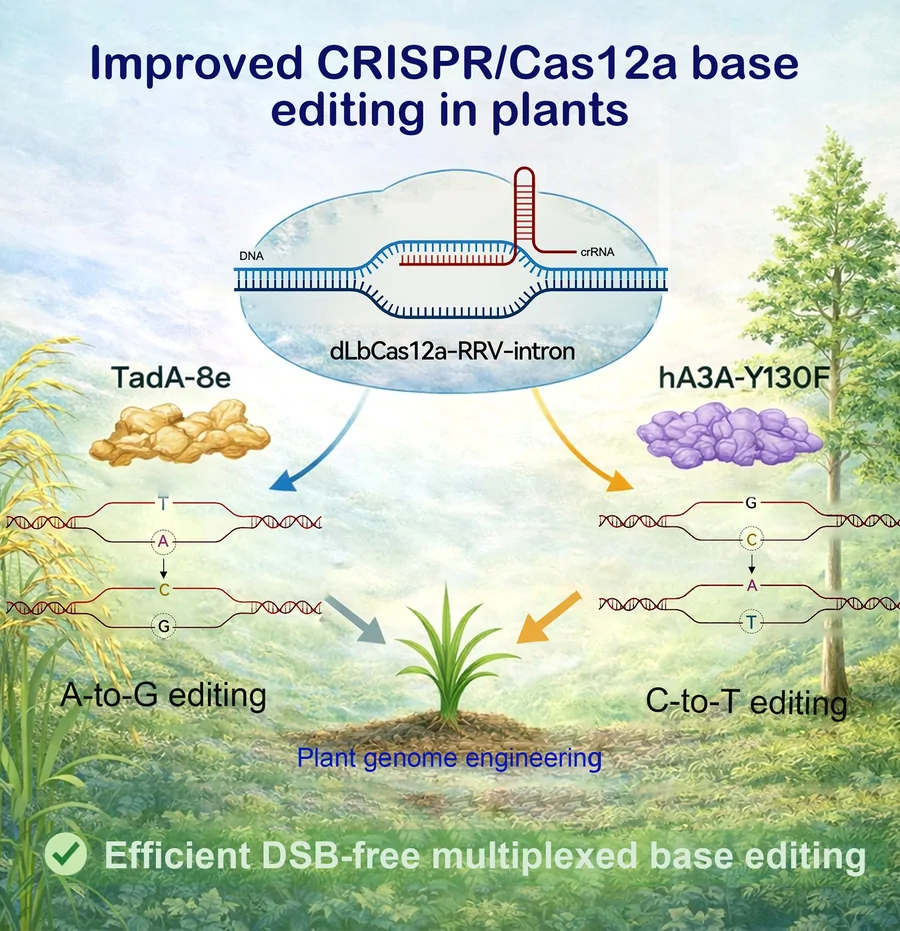

## INTRODUCTION

In this genome engineering era, many CRISPR‐Cas‐based genome editing tools have been developed to support plant research and crop improvement (Li et al., 2024a; Tuncel et al., 2025). Base editors such as cytosine base editors (CBEs) (Komor et al., 2016; Nishida et al., 2016) and adenine base editors (ABEs) (Gaudelli et al., 2017), due to their high precision and efficiency, are among the promising tools that have been rapidly adopted in many crops (Molla et al., 2021; Tuncel et al., 2025). Since the initial reports of CBEs in plants (Lu and Zhu, 2017; Zong et al., 2017), multiple versions of improved CBEs have been developed (Jin et al., 2020; Zeng et al., 2020; Ren et al., 2021a; Contiliani et al., 2025). Similarly, ABEs such as ABE7.10 have been demonstrated in many plant species including rice (Hua et al., 2018; Li et al., 2018). These base editors are powerful tools to introduce pinpoint mutations underlying various traits, such as herbicide resistance (Liu et al., 2020a; Molla et al., 2021; Tuncel et al., 2025). Compared to the prime editing systems, which can confer more precise sequence replacement (Anzalone et al., 2019; Lin et al., 2020), base editors generally exhibit higher editing efficiencies and can readily generate homozygous or biallelic point mutations within a single generation in many plant species (Li et al., 2021a; Wu et al., 2022a).

These base editors are built on the most widely used Class 2 type II‐A SpCas9 system, and specifically utilize a D10A mutation‐based nickase (Komor et al., 2016; Nishida et al., 2016; Gaudelli et al., 2017). However, genomic targeting by these base editors is constrained by the NGG protospacer adjacent motif (PAM) requirement of SpCas9. To broaden the targeting scope of base editing, scientists have harnessed different SpCas9 variants and Cas9 orthologs to develop base editors (Molla et al., 2021), such as Cas9‐NG targeting the NG PAM (Endo et al., 2019; Hua et al., 2019; Ren et al., 2019; Wang et al., 2019; Zhong et al., 2019; Zhang et al., 2021a), iSpyMacCas9 targeting the A‐rich PAM (Ren et al., 2021a; Sretenovic et al., 2021b), and near PAM‐less SpRY (Xu et al., 2021; Ren et al., 2021b; Li et al., 2021c). These additional base editors, albeit their capability for targeting alternative PAMs, often suffer from lower editing efficiency. In addition, all Cas9‐derived base editors are based on Cas9 nickases, which induce single‐strand DNA breaks. In CBEs, endogenous uracil‐DNA glycosylase activity is inhibited by uracil glycosylase inhibitor (UGI) that is fused to the Cas9 nickase (Komor et al., 2016). Nevertheless, insertions and deletions (indels) are frequent byproducts (Komor et al., 2016; Ren et al., 2021a), indicating the formation of DNA double‐strand breaks (DSBs). Furthermore, in a multiplexed editing setting, concurrent DNA nicks or DSBs may trigger undesired chromosomal rearrangements, which could only be revealed by long‐read, high‐coverage whole genome sequencing. It is hence very desirable to develop high‐efficiency, high‐purity, and DNA break‐free base editors for highly multiplexable base editing in plants.

Class 2 type V‐A Cas12a nucleases are also widely used for genome editing in plants (Zhang et al., 2019; Tuncel et al., 2025). Unlike SpCas9, Cas12a nucleases recognize T‐rich PAMs and require only CRISPR RNA (crRNA) for DNA targeting and cleavage (Zetsche et al., 2015). Multiplexed editing can be conveniently achieved by different crRNA expression and processing systems, whether based on the RNase activity of Cas12a for self‐processing the crRNA array (Fonfara et al., 2016; Wang et al., 2017), or based on the precise dual hammerhead (HH)‐hepatitis delta virus (HDV) ribozyme system (Tang et al., 2017; Zhang et al., 2021b). Among all Cas12a orthologs reported, LbCas12a is most popular for genome editing in plants due to its relatively high activity (Zhang et al., 2019; Tuncel et al., 2025). A series of LbCas12a variants have been engineered to confer high activity in ambient temperatures for genome editing in plants, such as ttLbCas12a that carries a D156R mutation (Kleinstiver et al., 2019; Schindele and Puchta, 2019), LbCas12a–E795L (Zhang et al., 2022), LbCas12a stacked with D156R and E795L mutations (Xin et al., 2024), hyperCas12a (Guo et al., 2022), and LbCas12a‐RV and RRV variants (Zhang et al., 2023c). Given the unique characteristics of CRISPR‐Cas12a, it is appealing to develop base editors from these systems to expand targeting scope and accommodate highly multiplexed editing while maintaining chromosomal integrity.

However, the development of CRISPR‐Cas12a base editors has been challenging because appropriate Cas12a nickases have not been engineered. Rather, nuclease‐deactivated Cas12a (dCas12a) proteins are used, which greatly compromise base editing efficiency. For example, fusion of an adenine deaminase to dLbCas12a led to very low base editing efficiency, ~0.45%, in cotton (Wang et al., 2022). Major breakthroughs were made in 2023 when three independent groups reported successful development of Cas12a CBEs and ABEs with double‐digit editing efficiencies when the ttLbCas12a variant and high‐activity deaminases were used (Cheng et al., 2023; Gaillochet et al., 2023; Wang et al., 2023). Furthermore, we and others have shown that linker optimization is necessary to obtain high‐efficiency Cas12a CBEs and ABEs for genome editing in rice, maize, and wheat (Cheng et al., 2023; Gaillochet et al., 2023). Despite these successes, current Cas12a CBEs are still not very robust and efficient to readily generate homozygous or biallelic base edits in rice (Cheng et al., 2023; Gaillochet et al., 2023; Wang et al., 2023), and challenges are expected when applying these tools in other plant species. Previously, we showed that highly multiplexed genome editing by LbCas12a generated chromosomal rearrangements due to concurrent DNA cleavages (Zhang et al., 2023d). It is envisioned that multiplexed base editing based on the Cas9 nickase system may also promote chromosomal recombination, thereby generating similar issues. Hence, developing efficient dCas12a base editors without generating DNA breaks may provide a safer practice for multiplexed base editing applications in plants. This is one of our motivations for this study.

Based on an artificial protein evolution pipeline, we recently developed multiple promising LbCas12a variants that confer high genome editing activity in human cells and plants, including LbCas12a‐RV, LbCas12a‐RVQ, and LbCas12a‐RRV (Zhang et al., 2023c). For example, LbCas12a‐RRV showed higher target DNA‐binding activity and consistently outperformed the wild‐type (WT) LbCas12a and ttLbCas12a in rice, tomato, and poplar (Zhang et al., 2023c). Similar to the observation with SpCas9 (Grutzner et al., 2021), insertion of multiple introns into the ttLbCas12a coding sequence was found to improve genome editing efficiency in Arabidopsis (Schindele et al., 2023). Inspired by these reports, we set out to explore the strategies of new LbCas12a variants and introns for the development of the next‐generation Cas12a CBEs and ABEs. By testing the systems using stable transformation of rice (an annual monocot) and poplar (a perennial dicot), we hope to achieve promising results that can be generally applicable to all plants.

## RESULTS

### dLbCas12a‐RRV base editors enable precise *OsACCase* editing to generate herbicide‐resistant rice

**Figure 1:**
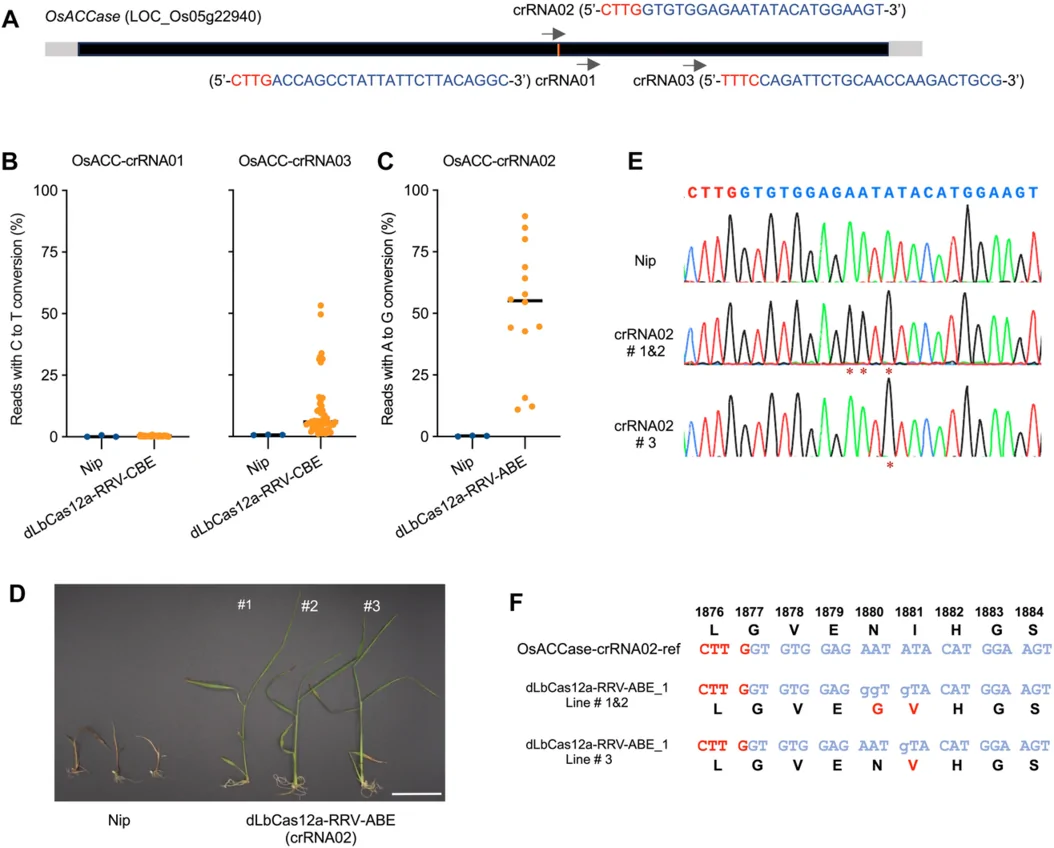
**Engineering herbicide resistance by dLbCas12a‐RRV mediated base editing of** ***OsACCase*** **(A)** Schematic representation of the *OsACCase* genomic region and the dLbCas12a‐RRV target sites used for base editing. Three guide RNAs were designed to introduce C‐to‐T or A‐to‐G conversions within the coding sequence of *OsACCase* to generate herbicide‐resistant alleles, and the corresponding PAM sequences are highlighted in red. **(B**, **C)** Quantification of C‐to‐T **(B)** and A‐to‐G **(C)** editing frequency in independent T_0_ rice lines. Each dot represents an individual T_0_ event, and the editing percentages were determined by NGS. **(D)** Haloxyfop selection of T_1_ progeny derived from base‐edited *OsACCase* lines. Plants were grown on the media with 2.66 μM haloxyfop. Representative surviving T_1_ events (1–3) exhibit normal growth, whereas wild‐type seedlings fail to survive and show severe chlorosis and growth arrest. **(E**, **F)** Genotyping of herbicide‐resistant T_1_ plants. Sanger sequencing chromatograms confirming the presence of the desired A‐to‐G conversion (indicated by an asterisk) in Haloxyfop‐resistant T_1_ lines in **(E)**, and the amino acid changes resulting from base substitutions are indicated in **(F)**. Wild‐type Nipponbare (Nip) seedlings or genomic DNA were used as the negative control in **(B**–**E)**.

Since LbCas12a‐RRV outperformed ttLbCas12a in genome editing in plants (Zhang et al., 2023c), we decided to develop new CBE and ABE based on the LbCas12a‐RRV variant. We wanted to test these Cas12a base editors for developing herbicide resistance in rice, as trait engineering has not been previously demonstrated in crops with Cas12a base editors. To introduce herbicide‐resistance mutations into rice, we designed three guide RNAs targeting the *OsACCase* coding sequence, following previously reported herbicide‐resistance alleles (Figure 1A). These guides were cloned into dLbCas12a‐RRV cytosine or adenine base editor constructs using the deaminase configurations that we previously reported (Cheng et al., 2023).

To evaluate their potential for generating precise single‐nucleotide substitutions, we produced T_0_ plants for each construct and assessed editing efficiencies in these plants using amplicon‐based next‐generation sequencing (NGS). Across the three crRNAs, C‐to‐T editing at *OsACCase* occurred at substantially lower frequencies (Figure 1B). In contrast, A‐to‐G base editing mediated by the dLbCas12a‐RRV‐ABE showed high activity, with crRNA02 achieving up to 51.87% editing efficiency (Figure 1C), consistent with previous findings that the LbCas12a‐based ABEs display more robust editing than CBEs (Cheng et al., 2023; Gaillochet et al., 2023). These results demonstrate that dLbCas12a‐RRV can efficiently mediate adenine deamination at the *OsACCase* locus.

To test whether the edited alleles confer herbicide tolerance, we treated T_1_ progeny derived from edited lines with the ACCase‐inhibiting herbicide haloxyfop. Wild‐type Nipponbare seedlings exhibited chlorosis and failed to grow, whereas multiple T_1_ plants carrying dLbCas12a‐RRV‐ABE‐edited *OsACCase* alleles exhibited normal growth (Figure 1D). Genotyping of herbicide‐resistant T_1_ plants by Sanger sequencing confirmed the presence of the expected single‐nucleotide substitutions. Representative Sanger sequencing chromatograms showed clean and heritable A‐to‐G conversions in T_1_ individuals (Figure 1E). Allelic annotation further revealed the precise A‐to‐G mutations at the crRNA02 target site (Figure 1F), consistent with the predicted amino acid changes associated with ACCase‐inhibitor resistance. Together, these results establish dLbCas12a‐RRV‐BEs as efficient tools for generating herbicide‐resistant *OsACCase* alleles and demonstrate their utility for engineering agronomically valuable traits in rice.

### dLbCas12a‐RRV–intron‐CBE markedly enhances C‐to‐T editing efficiency in rice

**Figure 2:**
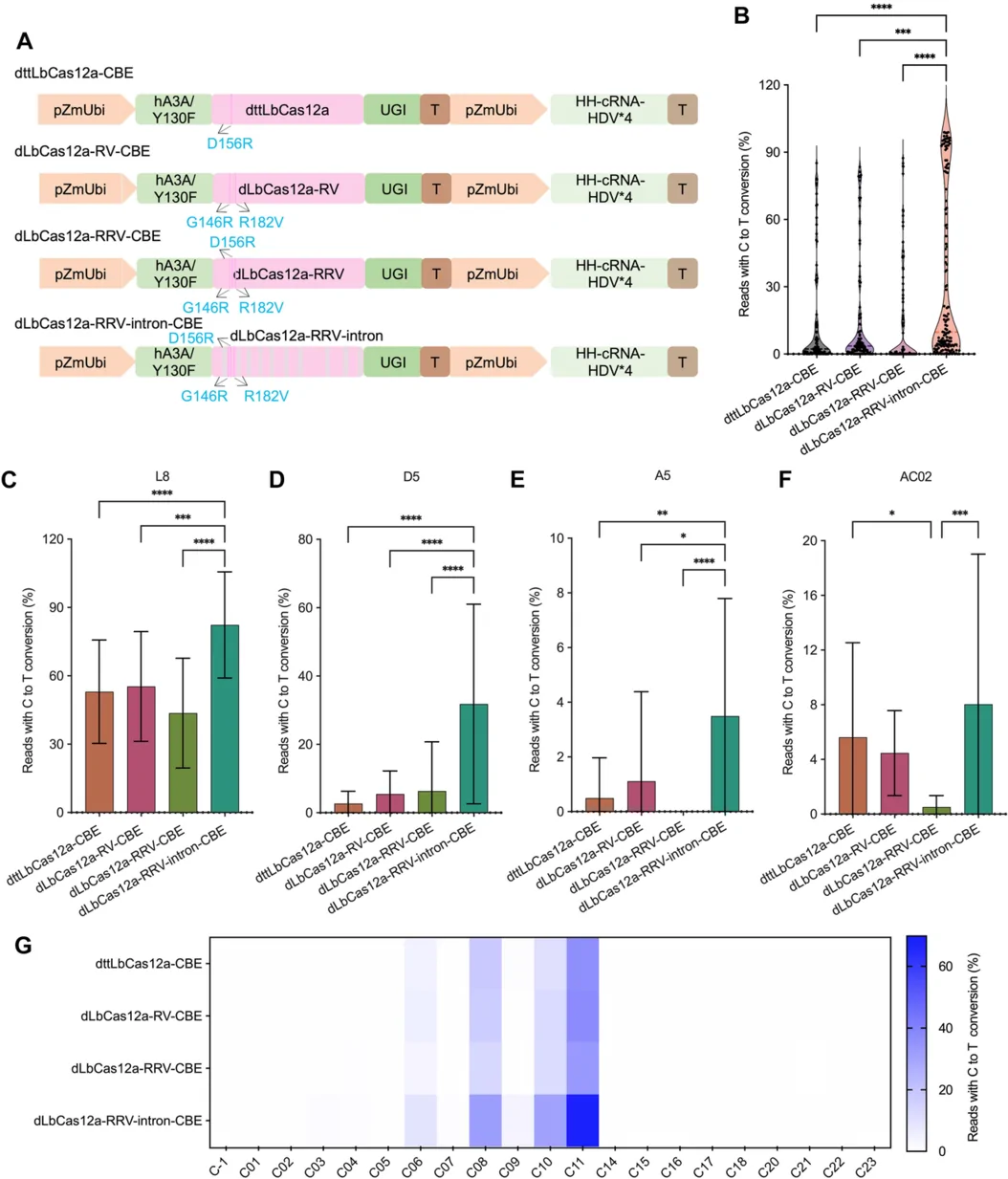
Comparison of four dLbCas12a‐based C‐to‐T cytosine base editors in rice T_0_ lines **(A)** Schematic illustrating diverse dLbCas12a variant‐based cytosine base editors (CBEs) driven by *ZmUbi1* promoter and using a tandem HH‐crRNA‐HDV system for multiplexed editing. **(B)** Violin plots showing C‐to‐T conversion rates across four sites with canonical TTTV PAMs. The distribution width reflects the density of editing frequencies across all sequenced lines. Each dot represents an independent T_0_ rice line. The median and quartiles are indicated by dotted lines. **(C**–**F)** Quantification of C‐to‐T conversion at individual target sites L8 **(C)**, D5 **(D)**, A5 **(E)**, and AC02 **(F)**. Error bars represent the standard deviation across individual T_0_ events. **(G)** Heatmaps display cytosine base‐editing working windows across four tested sites. *P*‐values were obtained using one‐tailed Student's *t*‐test, *P < 0.05, **P < 0.01, ***P < 0.001, ****P < 0.0001.

Although dLbCas12a‐based base editors have expanded the genome editing toolkit in plants, their efficiency, particularly for cytosine base editing, remains suboptimal at many endogenous loci. For example, our initial editing of *OsACCase* using dLbCas12a‐RRV‐CBE produced only modest C‐to‐T substitutions (Figure 1B), indicating that further optimization is required. Previous work in Arabidopsis showed that intron incorporation can enhance ttLbCas12a editing efficiency (Schindele et al., 2023), motivating us to test engineered dLbCas12a variants‐derived base editors in rice. To systematically evaluate the performance of enhanced LbCas12a variants, we constructed four dLbCas12a‐based cytosine base editors: dttLbCas12a‐CBE, dLbCas12a‐RV‐CBE, dLbCas12a‐RRV‐CBE, and dLbCas12a‐RRV–intron‐CBE (Figure 2A).

Among these four CBEs, dttLbCas12a‐CBE represents the state‐of‐the‐art Cas12a CBE prior to this study (Cheng et al., 2023), which is used for benchmarking new CBEs. dLbCas12a‐RV‐CBE and dLbCas12a‐RRV‐CBE are based on two LbCas12a variants that were shown to be overall more robust than ttLbCas12a based on our previous study (Zhang et al., 2023c). Since we hypothesized that dLbCas12a‐RRV‐CBE would be the most efficient one among the three CBEs, we focused on this CBE and generated an intron‐containing version for further comparison. Each Cas12a‐CBE was driven by the ZmUbi1 promoter and equipped with a multiplex HH‐crRNA‐HDV expression system enabling simultaneous expression of four crRNAs (Figure 2A). These editors were tested at four endogenous rice genomic sites (L8, D5, A5, and AC02), all containing canonical TTTV PAMs compatible with LbCas12a.

NGS analysis of T_0_ transformants revealed clear differences in C‐to‐T editing activity among the four variants. When aggregated across all target sites, the dLbCas12a‐RRV–intron‐CBE construct displayed the highest median C‐to‐T conversion efficiency and the widest activity distribution (0.14%–98.93%, average 32.73%) (Figure 2B). In contrast, dttLbCas12a‐CBE, dLbCas12a‐RV‐CBE, and dLbCas12a‐RRV‐CBE exhibited relatively moderate activity, with base editing efficiencies of 0.27%–85.22% (average 15.47%), 0.55%–83.47% (average 16.60%), and 0.13%–87.41% (average 12.63%), respectively.

At individual loci, dLbCas12a‐RRV–intron‐CBE outperformed all other variants. At the L8 site, dLbCas12a‐RRV–intron‐CBE generated the highest C‐to‐T editing efficiency, with significantly greater activity relative to the other editors (Figure 2C). A similar enhancement was observed at D5, where dLbCas12a‐RRV–intron‐CBE produced robust C‐to‐T conversions that were largely absent in the dttLbCas12a, RV, and RRV constructs (Figure 2D). Even at the relatively low‐efficiency A5 and AC02 sites, dLbCas12a‐RRV–intron‐CBE remained the most active variant, generating significantly higher editing frequencies than the other editors (Figure 2E, F). To further characterize the editing profiles, we mapped cytosine editing windows across all four loci. Heatmap visualization revealed that four dLbCas12a‐based CBEs possessed a similar active deamination window from C6 to C11, with enriched editing at central protospacer positions (Figure 2G).

Given the enhanced editing activity observed at individual targets, we next examined whether these improvements translated into increased co‐editing across multiple loci within the individual T_0_ rice line. Analysis of multiplex editing outcomes revealed that all four CBEs can generate edits at least at one target site. Notably, dLbCas12a‐RRV–intron‐CBE produced a higher proportion of rice lines harboring simultaneous edits at two to four target sites among the four tested loci, indicating improved co‐editing capacity relative to the other variants (Figure S1).

**Figure 3:**
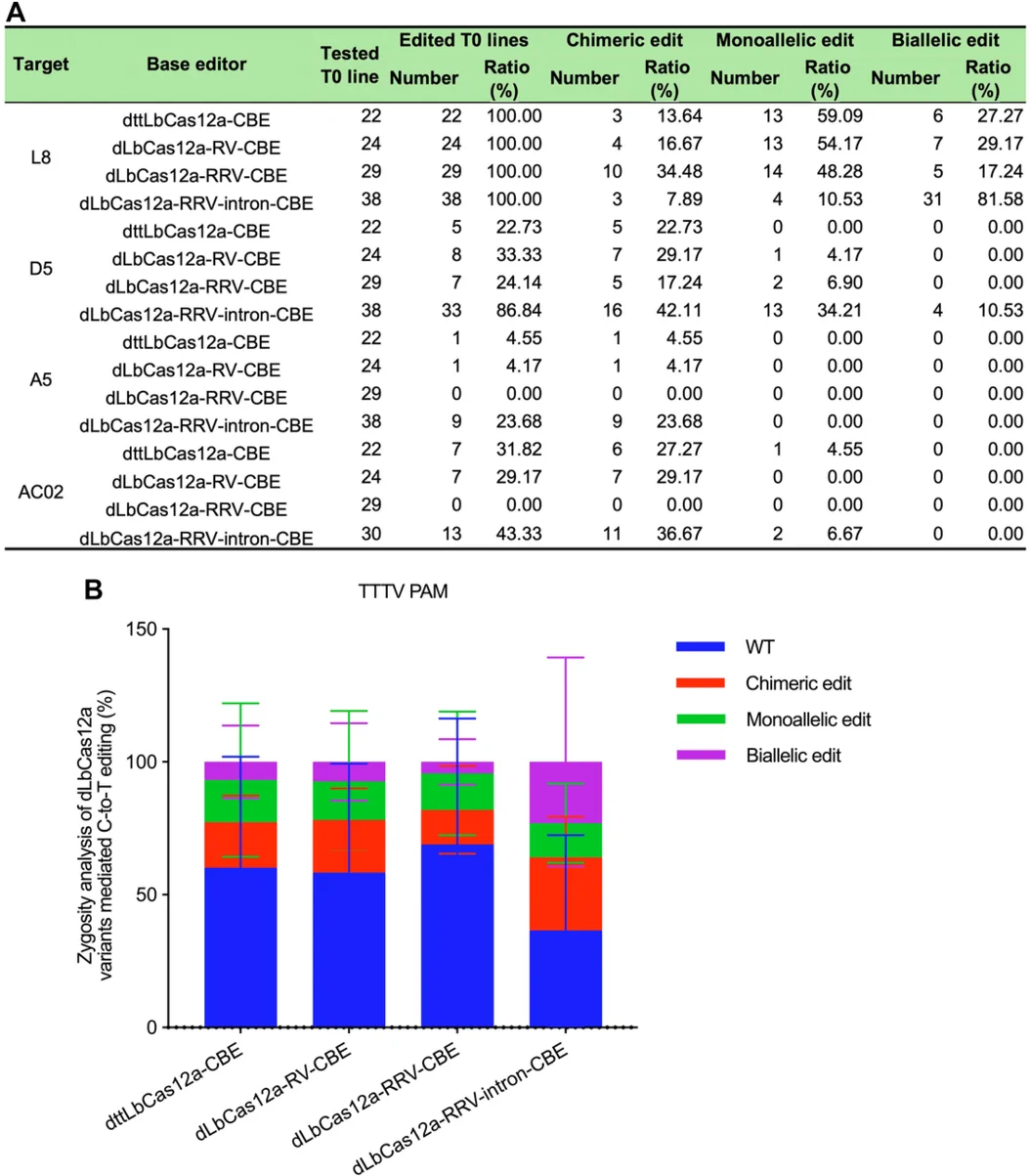
Genotypes of T₀ rice plants edited by dLbCas12a variant‐based cytosine base editors **(A)** Summary of C‐to‐T editing frequencies at four endogenous target sites in rice T_0_ plants edited by the indicated dLbCas12a variant‐derived cytosine base editors (CBEs). **(B)** Zygosity classification of editing outcomes generated by the four CBE variants. T_0_ plants were categorized based on their C‐to‐T editing frequencies as follows: < 5%, wild‐type (WT); 5%–30%, chimeric edit; 30%–75%, monoallelic edit; > 75%, biallelic edit. Mutation frequencies were determined by NGS of PCR amplicons, and all sequencing data were analyzed using CRISPR RGEN tools.

We further conducted zygosity analysis across T_0_ individuals generated from the four multiplexed CBE constructs. As expected, dLbCas12a‐RRV–intron‐CBE generated the highest proportion of biallelic edits across the tested sites (Figure 3A). For example, this construct generated 81.58% biallelic editing at the L8 site, compared with 17.24% to 29.17% for the other three variants (Figure 3A). At the D5 site, only dLbCas12a‐RRV–intron‐CBE induced biallelic edits (4 out of 38 lines; 10.53%) (Figure 3A). Summary of the zygosity analysis of all four target sites showed an overall trend that dLbCas12a‐RRV–intron‐CBE produced more biallelic editing (Figure 3B).

Together, these data identify dLbCas12a‐RRV–intron‐CBE as the most efficient and broadly applicable C‐to‐T editor among the variants tested, representing a substantially improved dLbCas12a‐based CBE tool across efficiency, robustness, multiplex editing capability, and biallelic edits for rice genome engineering.

### dLbCas12a‐RRV–intron‐ABE shows detectable improvement in A‐to‐G editing in rice

**Figure 4:**
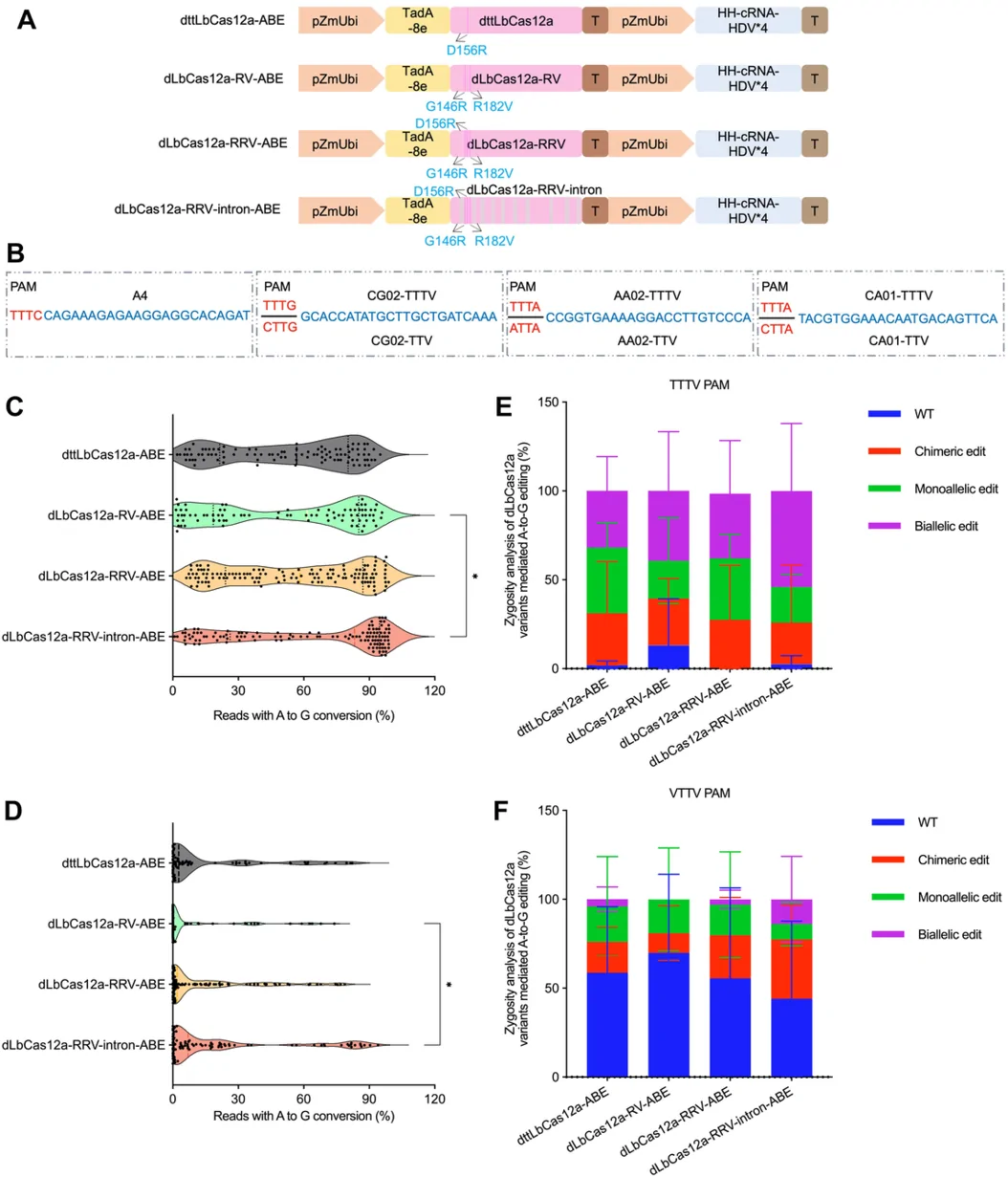
Comparison of four dLbCas12a‐based adenine base editors in rice T_0_ lines **(A)** Schematic representation of diverse dLbCas12a variant‐based ABEs driven by *ZmUbi1* promoter and using a tandem HH‐crRNA‐HDV system for multiplexed editing. **(B)** The crRNA spacers used for dLbCas12a‐based A‐to‐G editing in rice. Three crRNAs targeting genomic sites bearing both TTTV and VTTV PAMs at different rice loci, and one crRNA (A4) only targeting a single TTTV‐PAM site. PAM sequences are highlighted in red, and crRNA spacers are shown in blue. **(C**, **D)** Validation of four dLbCas12a‐based A‐to‐G editing frequency at target sites with TTTV PAM **(C)** and VTTV PAM **(D)** in T_0_ rice plants. Data are shown as violin plots, with distribution width representing density of editing frequencies. Each dot represents an independent T_0_ rice line. The median and quartiles are indicated by dotted lines. **(E**, **F)** Zygosity classification of editing outcomes generated by the four ABE variants at TTTV PAMs **(E)** and VTTV PAMs **(F)**. T_0_ plants were categorized based on their A‐to‐G editing frequencies as follows: < 5%, wildtype (WT); 5%–30%, chimeric edit; 30%–75%, monoallelic edit; > 75%, biallelic edit. Mutation frequencies were determined by NGS of PCR amplicons. *P*‐values were obtained using one‐tailed Student's *t*‐test, *P < 0.05.

Similarly, we generated and evaluated four TadA8e‐fused LbCas12a‐based ABEs: dttLbCas12a‐ABE, dLbCas12a‐RV‐ABE, dLbCas12a‐RRV‐ABE, and dLbCas12a‐RRV–intron‐ABE (Figure 4A). Four crRNA spacers were evaluated in total: Three crRNAs that target genomic sites bearing both TTTV and VTTV PAMs at different rice loci, and one crRNA (A4) that targets only a single TTTV PAM site (Figure 4B). These four crRNAs were co‐expressed using the HH‐crRNA‐HDV expression array for multiplexed editing (Figure 4A).

Amplicon NGS analyses of T_0_ plants demonstrated that all four editors were capable of efficient A‐to‐G conversion at TTTV PAM sites, with average editing efficiencies ranging from approximately 52.38% to 63.25% across all tested sites (Figure 4C). dLbCas12a‐RRV–intron‐ABE displayed slightly higher editing activity at several TTTV targets (up to 99.09%, average 63.25%), whereas dttLbCas12a‐ABE, dLbCas12a‐RV‐ABE, and dLbCas12a‐RRV‐ABE produced slightly lower editing levels. Statistical comparisons revealed that only the difference between dLbCas12a‐RV‐ABE and dLbCas12a‐RRV–intron‐ABE reached significance, indicating that although the RRV‐intron variant trends higher, the overall variation among the four ABE constructs remains modest. At VTTV PAM sites, where editing frequencies were generally lower than at TTTV sites, all four editors remained active, with average editing efficiencies ranging from 11.03% to 22.12% across tested sites (Figure 4D). Again, dLbCas12a‐RRV–intron‐ABE showed a trend toward higher A‐to‐G conversion, but only the comparison with the RV variant was statistically significant.

We next assessed whether the different ABEs supported efficient co‐editing across multiple loci within the same T_0_ rice line. The results showed that all four ABE constructs could generate edits at two or more target sites (Figure S2). Notably, dLbCas12a‐RRV–intron‐ABE produced rice lines harboring simultaneous edits at four or more target sites among the seven tested loci. It generated a higher frequency of lines with six to seven simultaneously edited sites compared with the other dLbCas12a‐based ABEs, indicating enhanced co‐editing capacity (Figure S2). Importantly, among the seven tested sites, three harbored VTTV PAMs, which generally show lower editing efficiencies than canonical TTTV PAMs. Therefore, the observed co‐editing performance suggests that we would have achieved a higher level of multiplex co‐editing if we targeted only the canonical TTTV PAM sites.

To further assess editing performance, we analyzed zygosity distributions in T_0_ individuals. dLbCas12a‐RRV–intron‐ABE generated a substantially higher proportion of biallelic edits than the other variants at both TTTV and VTTV PAM sites (Figure 4E, F). Across the seven loci tested, dLbCas12a‐RRV–intron‐ABE showed the highest overall editing activity and generated the greatest proportion of biallelic T_0_ plants, except at the site AA02‐VTTV (Figure S3). These zygosity outcomes provide further confirmation that the intron‐containing LbCas12a‐RRV variant improves editing penetrance in T_0_ rice plants.

We further examined the editing windows of dLbCas12a‐based ABEs across all tested loci. Editing was concentrated primarily within the central region of the protospacer, with the strongest A‐to‐G conversions observed between protospacer positions A8–A16, with a preference for A12 (Figure S4A, B), and was similar across variants.

Together, these results show that all four dLbCas12a‐derived ABEs possess strong A‐to‐G editing capabilities in rice across both TTTV and VTTV PAM sites, consistent with our previous report (Cheng et al., 2023). Although the magnitude of improvement is modest compared with the dramatic gains observed for dLbCas12a‐RRV–intron‐CBE, dLbCas12a‐RRV–intron‐ABE reproducibly increased both editing frequency and the proportion of biallelic edits, indicating a measurable improvement.

### Evaluation of dLbCas12a‐ and its variant‐derived cytosine and adenine base editors in poplar

**Figure 5:**
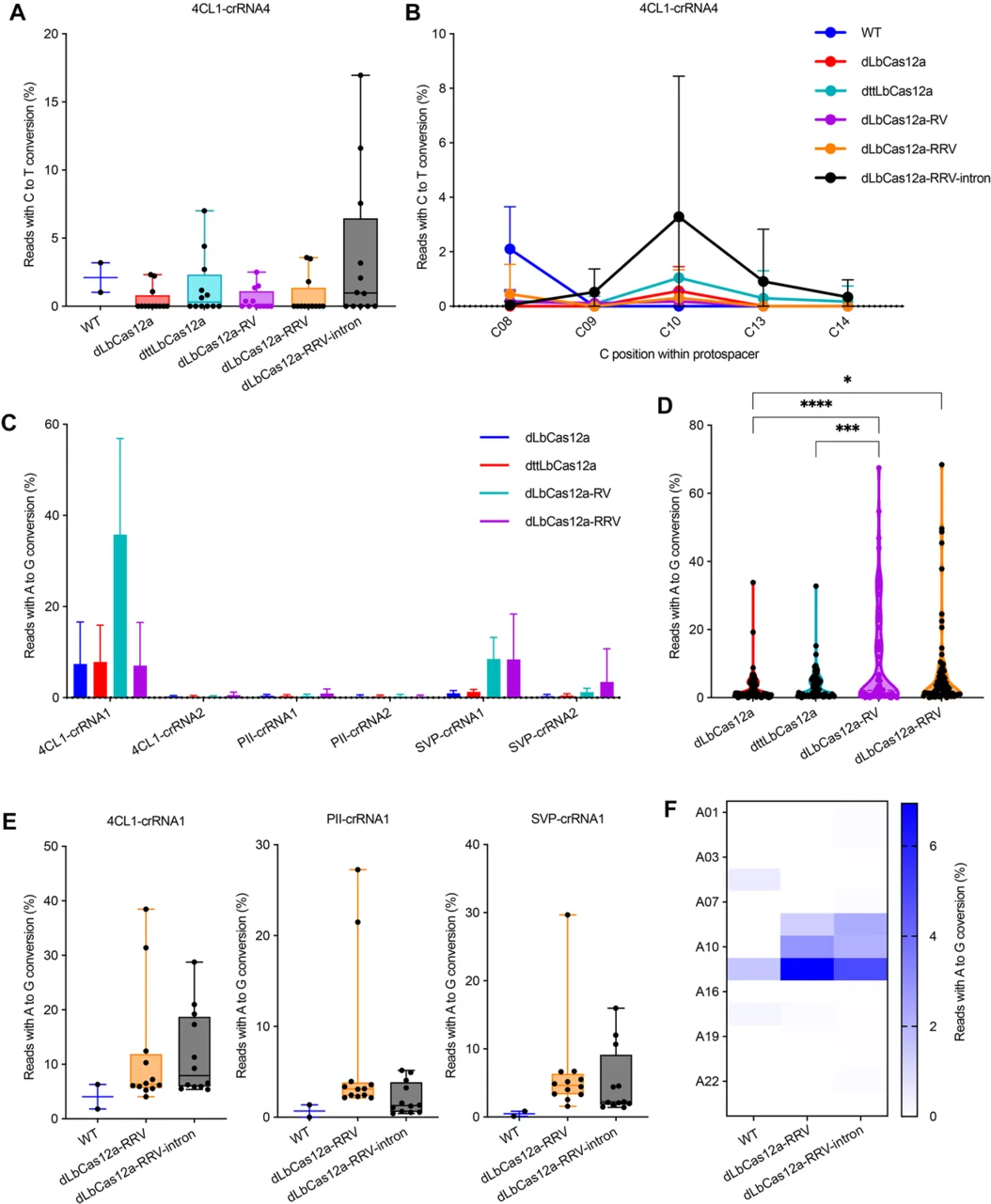
Assessing dLbCas12a and its variant‐based BEs in poplar T_0_ lines **(A**, **B)** Validation of five dLbCas12a‐CBEs mediated C‐to‐T editing frequency **(A)** and editing window **(B)** at target 4CL1‐crRNA4. **(C)** A‐to‐G editing activities of four dLbCas12a‐based adenine base editors (ABEs) at six endogenous poplar loci (4CL1‐crRNA1, 4CL1‐crRNA2, PII‐crRNA1, PII‐crRNA2, SVP‐crRNA1, and SVP‐crRNA2). Each bar represents the mean A‐to‐G conversion frequency from independent T_0_ plants. The data were shown as means ± *SD*. **(D)** Violin plots summarizing A‐to‐G base‐editing efficiency across all six target sites. Each dot corresponds to an independent poplar T_0_ transgenic line, and the distribution width reflects the density of editing frequencies. Statistical significance was determined using one‐tailed Student's *t*‐test (*P < 0.05, ***P < 0.001, ****P < 0.0001). **(E)** Bulked comparison of A‐to‐G conversion frequencies mediated by dLbCas12a‐RRV and its intron version at site 4CL1‐crRNA1, PII‐crRNA1, SVP‐crRNA1. **(F)** Heatmaps illustrating A‐to‐G editing windows for dLbCas12a‐RRV‐ABE and dLbCas12a‐RRV–intron‐ABE.

To determine whether dLbCas12a‐derived editors retain activity in a woody perennial, we evaluated cytosine and adenine base editing in poplar T_0_ plants. In addition to the four CBEs tested in rice, we also included the CBE based on WT LbCas12a. Six genomic loci were initially selected for CBE testing. Among these, only 4CL1‐crRNA4 showed detectable C‐to‐T editing, whereas the other five sites exhibited little or no activity (data not shown). At this locus, all five CBE variants produced low but measurable C‐to‐T conversions (average 0.47%–3.61%), with dLbCas12a‐RRV–intron‐CBE showing slightly higher efficiencies than the other variants (Figure 5A). Editing windows were narrow and confined to position C10, with modest broadening for dLbCas12a‐RRV–intron‐CBE (Figure 5B), which could be attributed to its higher activity.

Adenine editing was examined in two stages. In the first batch, four ABEs (dLbCas12a‐ABE, dttLbCas12a‐ABE, dLbCas12a‐RV‐ABE, and dLbCas12a‐RRV‐ABE) were tested across six endogenous loci (*4CL1*, *PII*, and *SVP*, two sites per gene). All constructs generated detectable A‐to‐G conversions, although efficiencies remained low (Figure 5C). Notably, dLbCas12a‐RV‐ABE and dLbCas12a‐RRV‐ABE showed the highest median editing levels (Figure 5D), compared to dLbCas12a‐ABE and dttLbCas12a‐ABE, indicating better performance of the LbCas12a‐RV and LbCas12a‐RRV variants, consistent with the previously reported plant genome editing results with these variants (Zhang et al., 2023c).

To compare the effects of intron insertion, a second batch tested only dLbCas12a‐RRV–intron‐ABE alongside dLbCas12a‐RRV‐ABE, with dLbCas12a‐RRV‐ABE intentionally repeated to enable cross‐batch comparison. Across three representative loci (4CL1‐crRNA1, PII‐crRNA1, and SVP‐crRNA1), both editors produced similar and modest A‐to‐G editing with average editing frequency ranging from 0.49% to 11.87% (Figure 5E). The intron‐containing RRV variant did not show improved editing relative to either RRV‐ABE or the previously tested RV‐ABE. Editing windows remained narrow (A08–A11), with no substantial expansion observed in the intron‐containing construct (Figure 5F).

Together, these results indicate that dLbCas12a‐derived CBEs and ABEs are functional in poplar but operate at substantially lower efficiencies and with narrower editing windows than in rice. Across both editing types, dLbCas12a‐RV, RRV, and RRV‐intron variants show modest but consistent improvements compared to dLbCas12a and dttLbCas12a, suggesting that these engineered LbCas12a variants remain beneficial in woody perennials, although further optimization will be required to achieve robust Cas12a‐mediated base editing in poplar.

### dLbCas12a‐RRV–intron‐based ABE and CBE induce genome‐wide mutations but no detectable crRNA‐dependent off‐target effects

To date, genome‐wide off‐target effects of Cas12a BEs have not been reported. To investigate this, we conducted whole‐genome sequencing (WGS) of three T_0_ plants edited by dLbCas12a‐RRV–intron‐CBE, three T_0_ plants edited by dLbCas12a‐RRV–intron‐ABE, along with three T_0_ plants transformed with GFP as controls. These edited lines were selected because they exhibited robust multiplex editing across tested endogenous loci (Figures S5, S6). Detailed NGS profiles confirmed efficient and precise C‐to‐T (Figure S5A–C) or A‐to‐G (Figure S6A–C) conversions at tested sites, ensuring that each WGS sample reflected active base‐editing outcomes suitable for off‐target analysis. Among the three selected plants edited by dLbCas12a‐RRV–intron‐ABE, line # 20 plant achieved simultaneous editing at all seven target sites (Figure S5B).

**Figure 6:**
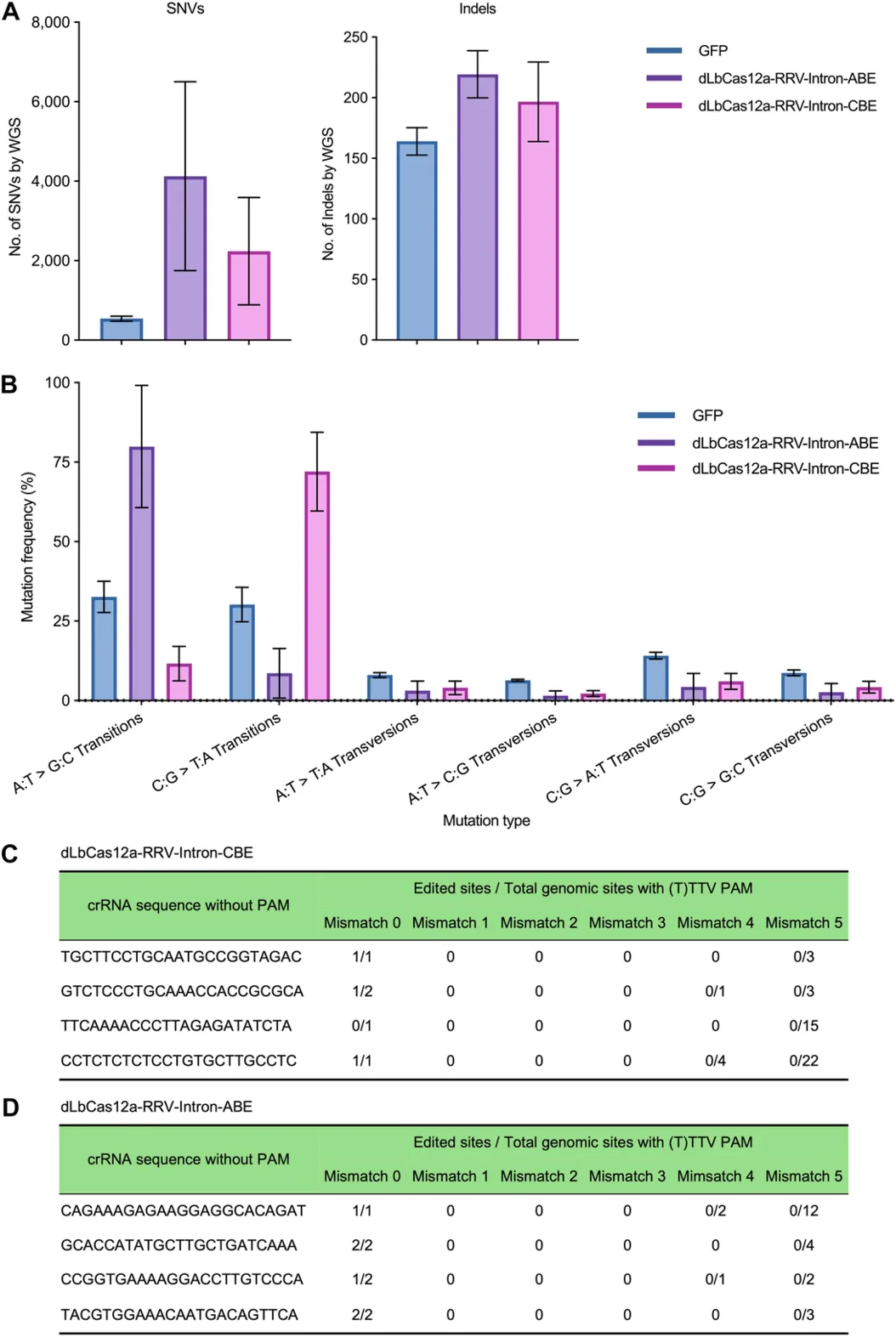
Whole‐genome sequencing (WGS)‐based off‐target analysis of the dLbCas12a‐RRV–intron mediated C‐to‐T and A‐to‐G editing in rice T_0_ lines **(A**, **B)** Genome‐wide evaluation of single‐nucleotide variants (SNVs) and insertions/deletions (indels) **(A)**, and base substitutions **(B)**. Error bars indicate the standard deviation. **(C)** Evaluation of crRNA‐specific off‐target effects in rice T_0_ lines for dLbCas12a‐RRV–intron‐CBE. **(D)** Evaluation of crRNA‐specific off‐target effects in rice T_0_ lines for dLbCas12a‐RRV–intron‐ABE. The predicted number of off‐target sites was estimated using Cas‐OFFinder, allowing mismatches of up to five nucleotides for each target sequence **(C**, **D)**.

Our WGS analysis revealed that edited plants accumulated noticeably more single‐nucleotide variants (SNVs) than GFP control plants, and a similar increase was not observed for small insertions/deletions (indels) (Figure 6A). The elevated SNV counts highlight a measurable increase in genome‐wide off‐target mutations associated with the use of dLbCas12a‐RRV‐intron base editors. Examination of substitution signatures further supported this conclusion: Edited plants displayed distinct shifts in transition patterns, including enrichment of A‐to‐G or C‐to‐T substitutions consistent with deaminase‐associated off‐target events (Figure 6B). For example, around 75% of these SNVs are C‐to‐T mutations and A‐to‐G mutations for plants edited by dLbCas12a‐RRV–intron‐CBE and by dLbCas12a‐RRV–intron‐ABE, respectively (Figure 6B). These patterns confirm the presence of genome‐wide, crRNA‐independent off‐target mutagenesis introduced during base editing.

We next assessed potential crRNA‐dependent off‐target sites predicted by Cas‐OFFinder (up to five mismatches allowed). For the CBE system, none of the predicted off‐target sites exhibited detectable C‐to‐T editing in any sequenced T_0_ plant (Figure 6C). A similar absence of A‐to‐G mutations was observed at all predicted off‐target candidates for the ABE system (Figure 6D). Thus, despite the elevated genome‐wide mutation load, no evidence of sequence‐specific, crRNA‐dependent off‐target editing was detected.

Together, these results demonstrate that dLbCas12a‐RRV–intron base editors do not cause detectable crRNA‐dependent off‐target activity in rice but do introduce measurable, crRNA‐independent genome‐wide off‐target mutations, an important consideration for future optimization.

## DISCUSSION

In this study, we engineered improved Cas12a CBEs and ABEs for base editing in plants. The dLbCas12a‐RRV–intron‐derived CBE and ABE were characterized and benchmarked against prior efficient ttLbCas12a‐CBE and ABE in rice and poplar. In rice, dLbCas12a‐RRV–intron‐CBE produced markedly higher C‐to‐T editing efficiencies across all tested loci, with dramatic gains in the proportion of biallelic edits in T_0_ plants, far exceeding those achieved by earlier Cas12a CBEs (2, 3). Our data are consistent with recent studies in wheat and barley (Lawrenson et al., 2024; Luo et al., 2025), suggesting that intron‐mediated enhancement of Cas12a expression and processing is broadly applicable across monocot species.

However, dLbCas12a‐RRV–intron‐ABE showed moderate improvement over the other ABEs. The different levels of improvement in CBEs and ABEs by new LbCas12a variants and the use of introns observed in rice could be largely attributed to baseline editing efficiency. For example, the dttLbCas12a‐ABE, developed in our previous study, is already as efficient as SpCas9‐based ABEs, readily generating multiplexed biallelic editing in rice (Cheng et al., 2023). This could be attributed to the use of a highly efficient TadA8e adenine deaminase, which is about 1,000 times more efficient than its previous version (Richter et al., 2020). Nonetheless, dLbCas12a‐RRV–intron‐ABE showed a reproducible increase in editing activity, generating a noticeably higher fraction of biallelic edits across both TTTV and VTTV PAMs in rice (Figures 4E, F, S3). Thus, even where per‐allele editing frequencies are near saturation, dLbCas12a‐RRV–intron‐ABE still enhances the editing outcomes in regenerated plants. In this work, we pioneered the use of Cas12a ABE to engineer a crop trait, specifically herbicide resistance in rice. It is expected that more traits can be engineered in many crops with these improved Cas12a base editors.

In poplar, the same LbCas12a variants function reliably but with reduced efficiency and narrower editing windows, highlighting species‐dependent constraints on Cas12a base editor activity. Notably, these LbCas12a variants, especially LbCas12a‐RRV, have previously been shown to be highly efficient nucleases in poplar (Zhang et al., 2023c). In addition, the A3A‐Y130F deaminase conferred efficient base editing in poplar when coupled with the Cas9D10A nickase (Li et al., 2021a). The introns used in this study were derived from Arabidopsis, and ttLbCas12a‐intron (codon‐optimized for Arabidopsis) have been shown to enhance genome editing in Arabidopsis (Schindele et al., 2023) and wheat (Luo et al., 2025). Collectively, all these individual components can work efficiently in poplar. Yet, the CBEs based on these LbCas12a variants, and their intron‐containing versions, display comparatively low base‐editing efficiencies in poplar. Although the precise underlying mechanism remains unclear, this reduced activity may be associated with an inherent characteristic of base editing with dCas12a proteins that do not induce DNA breaks.

There are strategies to be utilized for future improvement of such base editors to address low efficiency in poplar and other plants. First, tailored optimization of the codon usage and introns may help improve the CBE and ABE expression. Second, although the LbCas12a‐RRV variant is highly efficient, it may still be worth engineering and testing more promising variants. For example, previous research demonstrated improved Cas12a‐mediated base editing in human cells by using enCas12a (with D156R/G532R/K538R mutations) (Chen et al., 2022). Third, the use of high‐efficiency cytidine deaminases could help the development of better Cas12a CBEs. The efficient A3A‐Y130F cytidine deaminase sourced from humans was used in this study as well as in the development of Cas12a base editors in human cells (Chen et al., 2022). However, promising cytidine deaminases sourced from ocean mammals (Contiliani et al., 2025) or engineered by AI approaches (Fei et al., 2025) could be tested for potential enhancement of base editing efficiency. Fourth, the use of DNA‐binding proteins was shown to improve Cas9 base editors in human cells (Yin et al., 2023) and in plants (Zheng et al., 2024). Interestingly, this strategy was also found to help improve Cas12a base editors (Wang et al., 2023). Hence, this strategy may further help enhance the CBEs and ABEs developed in our study. Finally, the fact that intron‐containing dLbCas12a‐RRV CBE and ABE did not drastically improve the editing outcomes in poplar suggests there is room for optimization of this intron strategy on the basis of plant species.

Based on our data, the dLbCas12a‐RRV‐intron system already forms a desirable platform to engineer base editors for high activity in rice. So far, our study and other earlier studies have primarily focused on the development of Cas12a‐based CBEs and ABEs (Cheng et al., 2023; Gaillochet et al., 2023; Wang et al., 2023). However, other types of base editors have been developed based on the CRISPR‐Cas9 systems. For example, different dual base editors have been developed for introducing simultaneous C‐to‐T and A‐to‐G mutations, as useful tools for targeted gene evolution in plants (Li et al., 2020; Zhang et al., 2023a, 2023b; Fan et al., 2024; Zheng et al., 2024). By harnessing different DNA glycosylases, the CRISPR‐Cas9 base editing toolbox in plants was enriched with C‐to‐G base editors (Sretenovic et al., 2021a; Zeng et al., 2022; Jiang et al., 2025; Wu et al., 2025) and A‐to‐K base editors (Wu et al., 2023; Li et al., 2023b, 2024b). It can be envisioned that adopting these protein effectors to the dLbCas12a‐RRV‐intron platform would help engineer additional versatile Cas12a base editors capable of mediating transversion mutations in plants.

In recent years, whole‐genome sequencing has been used to evaluate genome‐wide off‐target effects of Cas9‐derived base editors in different crops such as rice (Jin et al., 2019; Ren et al., 2021a; Li et al., 2022; Wu et al., 2022b; Contiliani et al., 2025) and tomato (Randall et al., 2021; Sretenovic et al., 2023). The studies collectively revealed that genome‐wide gRNA‐independent off‐target mutations can be induced by highly active cytidine and adenine deaminases. Since we used such high‐efficiency deaminases to develop Cas12a base editors here, it is not surprising that we have detected gRNA‐independent off‐target mutations by WGS. However, we did not detect gRNA or crRNA‐dependent off‐target mutations, further supporting that CRISPR‐Cas12a is highly specific in plants (Tang et al., 2018) and the off‐target effects are due to the expression of the deaminases. Thousands of such off‐target mutations could be detected in the edited rice lines, and they drastically outnumbered somaclonal variation mutations due to tissue culture (Figure 6A, B). In the future, it is necessary to develop Cas12a base editors with minimal off‐target effects. Potential strategies may include split complementation of deaminases (Xiong et al., 2023) or embedding the deaminase within the Cas protein (Liu et al., 2020b).

In conclusion, we developed further improved Cas12a CBEs and ABEs by utilizing the LbCas12a‐RRV variant and intron. These new base editors will aid DNA break‐free multiplexed base editing in plants. The efficient LbCas12a‐RRV‐intron platform will promote further innovation in developing additional base editors for dual base editing or transversion base editing. This study also sheds light on future directions toward enhancing the Cas12a base editors while minimizing potential off‐target effects.

## MATERIALS AND METHODS

### Vector construction

The rice codon‐optimized pYPQ230 (Addgene #86210), pYPQ230‐D156R (Addgene #195366), pYPQ230‐RV (Addgene #188544), and pYPQ230‐RRV (Addgene #188543) vectors were used to generate the attL1‐attL5 Cas12a entry clones. Point mutations were introduced into pYPQ230 (LbCas12a), pYPQ230‐D156R (ttLbCas12a), pYPQ230‐RV (LbCas12a‐RV), and pYPQ230‐RRV (LbCas12a‐RRV) to generate dLbCas12a (D831A), dttLbCas12a (D831A), dLbCas12a‐RV (D831A), and dLbCas12a‐RRV (D831A) using the NEBuilder HiFi DNA Assembly Kit (New England Biolabs). For the construction of dLbCas12a‐RRV‐intron, the ttLbCas12a‐intron fragment was amplified from pDe‐ttLbCas12a‐i (Addgene #192453) and used to replace the LbCas12a region in pYPQ230, producing pYPQ230‐D156R‐intron. Subsequently, the G146R, R182V, and D831A substitutions were introduced to obtain dLbCas12a‐RRV‐intron.

Cas12a‐based base‐editing vectors were generated by replacing the dttLbCas12a region of dpYPQ230‐D156R‐CBE4 (Addgene #195369) or dpYPQ230‐D156R‐ABE5 (Addgene #195371) with dLbCas12a‐RV, dLbCas12a‐RRV, or dLbCas12a‐RRV‐intron fragments between *Xba*I and *Nco*I. Primers used in this study are listed in Table S1.

Single‐stranded oligonucleotides for all target sites (Table S2) were synthesized by GeneWiz. For expression of four crRNAs in attL5‐attL2 entry vectors, a tandem HH‐crRNA‐HDV architecture was adopted as previously described (Zhang et al., 2021b). Annealed protospacers were individually cloned into pYPQ131‐STU‐Lb (Addgene #138096), pYPQ132‐STU‐Lb (Addgene #138099), pYPQ133‐STU‐Lb (Addgene #138102), and pYPQ134‐STU‐Lb (Addgene #138105) via *Bsm*BI. The four crRNA cassettes were then assembled into the rice expression vector pYPQ144‐ZmUBI‐pT (Addgene #138108) using Golden Gate assembly.

For poplar, a six‐target crRNA system was constructed. Four crRNA protospacers were cloned into pYPQ131‐STU‐Lb (Addgene #138096), pYPQ132‐STU‐Lb (Addgene #138099), pYPQ133‐STU‐Lb (Addgene #138102), and pYPQ134‐STU‐Lb (Addgene #138105), and assembled into pYPQ144 (Addgene #69296). Two additional crRNAs were cloned into pYPQ131‐STU‐Lb and pYPQ132‐STU‐Lb and assembled into pYPQ142‐AtUBQ10. The six crRNA cassettes were combined by enzymatic digestion and ligation using *Nco*I/*Spe*I‐linearized pYPQ142‐AtUBQ10‐crRNAs and *Nco*I/*Xba*I‐linearized pYPQ144‐crRNAs with the Instant Sticky‐End Ligase Master Mix (New England Biolabs).

Final T‐DNA expression constructs were generated by three‐way Gateway LR recombination using the attR1‐attR2 destination vector pYPQ203 (Addgene #86207) for rice or pYPQ202 (Addgene #86198) for poplar, together with the attL5‐attL2 crRNA entry clone and the attL1‐attR5 base editor entry clone.

### Rice stable transformation

The Japonica cultivars Kitaake and Nipponbare were used for transformation. *Agrobacterium*‐mediated transformation followed the previous report (Zhang et al., 2021b). Surface‐sterilized seeds were placed on callus induction medium for 14 d, and healthy calli were excised for infection. T‐DNA constructs were introduced into *Agrobacterium tumefaciens* EHA105 (OD_600_ = 0.5, 100 μM acetosyringone). Calli were co‐cultivated with *Agrobacterium* for 3–5 d at 25°C in the dark and then washed thoroughly. Infected calli were selected on hygromycin‐containing medium (50 mg L^−1^) for 2 plus 2 weeks at 32°C under light. Actively growing white calli were transferred to regeneration medium I at 29°C (16 h/8 h light/dark), and regenerated shoots were moved to regeneration medium II. Leaf tissue from T_0_ plants was collected for DNA extraction and genotyping.

### Poplar stable transformation

Poplar (*Populus alba × tremula* clone 717‐1B4) was transformed following a previously described method (Li et al., 2023a). T‐DNA constructs were introduced into *Agrobacterium tumefaciens* strain GV3101. Regenerated shoots were selected on shoot‐induction medium supplemented with 20 μg mL^−1^ hygromycin. Shoots reaching 1–2 cm in length were transferred to root‐induction medium containing 20 μg mL^−1^ hygromycin. Rooted plantlets were propagated and subsequently used for genotyping.

### Mutation analysis by next‐generation sequencing

Amplicon deep sequencing was used to assess base‐editing outcomes in rice and poplar. Genomic DNA was extracted from leaf tissue of stable transgenic plants using the CTAB method (Stewart and Via, 1993). For next‐generation sequencing (NGS), amplicons were generated through two rounds of PCR. The first round used target‐specific primers containing bridge sequences. Two microliters of the first‐round product served as the template for the second round using Hi‐TOM barcoding primers (Liu et al., 2019). Approximately 20–30 barcoded amplicons (< 500 bp) were pooled, column‐purified, normalized to 20 ng μL^−1^, and combined to yield at least 500 ng total double‐stranded DNA. Libraries were sequenced by GeneWiz using the Illumina MiSeq. 2 × 250 bp platform. NGS data were analyzed using the CRISPR Rgen BE Analyzer (http://www.rgenome.net/be-analyzer/#!). Edited rice plants were classified into four genotype categories (wild‐type (WT), chimeric, monoallelic, and biallelic edit) based on their C‐to‐T or A‐to‐G editing frequencies, following previously described criteria with minor modifications (Pan et al., 2022; Cheng et al., 2023; Zhang et al., 2023c): < 5%, WT; 5%–30%, chimeric edit; 30%–75%, monoallelic edit; > 75%, biallelic edit.

For Sanger sequencing, target regions were amplified with locus‐specific primers. PCR products were treated with ExoI and Quick‐CIP (New England Biolabs), mixed with 5 μL of sequencing primer, and submitted to GeneWiz for sequencing. Chromatograms were analyzed using SnapGene.

### Herbicide treatment of rice plants

T_1_ rice seeds were germinated on 1/2MS medium in magenta boxes. After 5 d, seedlings of uniform height were selected and transferred to 1/2MS media supplemented with 2.66 μM of Haloxyfop‐R‐methyl. The phenotype was recorded after 15 d of transfer to herbicide containing medium. Genomic DNA was isolated from the leaves of surviving plants, and target loci genotyping was performed by Sanger sequencing.

### WGS and off‐target analysis

Genomic DNA extraction was performed using the DNeasy Plant Mini kit (QIAGEN, Germany). Sequencing of all plant samples was carried out by the Illumina® MiSeq® platform (Genewiz, U.S.). Wild‐type rice (*Oryza sativa* L.) Japonica cultivar Kitaake sequencing data were included in our analysis to help with variant filtering and calling (Gurel et al., 2023). The analysis pipeline followed an adapted protocol (Liu et al., 2021). Briefly, Quality control was conducted using FastQC (v0.11.9), and Skewer (v 0.2.2) was used to remove low‐quality reads (Jiang et al., 2014). Cleaned reads were mapped to the rice reference genome OsativaKitaake_499_v3.0 (Li et al., 2017) using BWA (0.7.17‐r1188) (Li and Durbin, 2009). BAM files were sorted with SAMtools (v1.10) (Li et al., 2009), and Picard (v 2.21.9) was utilized to mark duplicate reads. After pre‐processing BAM files, the Genome Analysis Toolkit (GATK, v3.8) was used for realignment and recalibration (McKenna et al., 2010). Variant detection was achieved with four different tools. Single Nucleotide Variants (SNVs) were identified using LoFreq (v2.1.2) (Wilm et al., 2012), MuTect2 (Benjamin et al., 2019), and VarScan (v2.4.6) (Koboldt et al., 2012), while indel calling was done with MuTect2, VarScan, and Pindel (v0.2.5b9) (Ye et al., 2009). Only SNVs and indels that were consistently identified by their respective tools were retained. BEDTools (v2.27.1) and BCFtools (v1.17) were used for filtering SNVs/indels and processing VCF files. The resulting mutations were visualized with IGV software. Cas‐OFFinder (Bae et al., 2014) was employed to detect genome‐wide off‐target sites, allowing up to 5‐bp mismatches, with IGV being used to double‐check potential off‐target sites. Figures were generated using Python's Matplotlib library.

## CONFLICTS OF INTEREST

The authors declare no conflicts of interest.

## AUTHOR CONTRIBUTIONS

Y.Q. and Y.C. designed the project. Y.C. generated the Cas12a CBE and ABE vectors. Y.C. conducted rice transformation and genotyping, with the help of D.W., G.L., and Y.C. conducted poplar transformation and genotyping. M.Z. conducted WGS data analysis. R.M. genotyped rice plants and tested them for herbicide resistance. Y.C. and Y.Q. wrote the manuscript, with input from all authors. All authors reviewed and approved the manuscript.

## Supporting information

Additional Supporting Information may be found online in the supporting information tab for this article: http://onlinelibrary.wiley.com/doi/10.1111/jipb.70249/suppinfo

**Figure S1.** Co‐editing outcomes of multiplex base editing mediated by dLbCas12a variant‐based cytosine base editors in T_0_ rice stable lines
**Figure S2.** Co‐editing outcomes of multiplex base editing mediated by dLbCas12a variant‐based adenine base editors in T_0_ rice stable lines
**Figure S3.** Zygosity classification of T_0_ rice plants edited by dLbCas12a variant‐based adenine base editors
**Figure S4.** The A‐to‐G editing window analysis of four dLbCas12a variant‐based A‐to‐G editors in rice T_0_ lines
**Figure S5.** Genotypes of T_0_ rice plants edited by dLbCas12a‐RRV–intron‐CBE for whole‐genome sequencing (WGS)
**Figure S6.** Genotypes of T_0_ rice plants edited by dLbCas12a‐RRV–intron‐ABE for whole‐genome sequencing (WGS)
**Table S1.** Oligos used in this study
**Table S2.** crRNAs used in this study

## Data Availability

Four newly developed Cas12a base editors, along with Golden Gate recipient and Gateway entry vectors for assembling multiplexed guide RNAs under the *AtUBQ10* promoter are made available at Addgene: dpYPQ230‐RRV‐ABE (Addgene #250716), dpYPQ230‐RRV‐CBE (Addgene #250717), dpYPQ230‐RRV–intron‐ABE (Addgene #250718), dpYPQ230‐RRV–intron‐CBE (Addgene #250719), pYPQ142‐AtUBQ10 (Addgene #250923), pYPQ143‐AtUBQ10 (Addgene #250985), and pYPQ144‐AtUBQ10‐pT (Addgene #250924). The whole‐genome sequencing (WGS) data have been deposited in the Sequence Read Archive (SRA) at the National Center for Biotechnology Information (NCBI), with accession numbers SRR30670647–SRR30670652 for genome‐edited samples and SRR30670612–SRR30670614 for GFP control samples.
