# Supplementary material for: Enhancing CRISPR‐Cas12a base editing in plants with LbCas12a variants and introns
Source: J Integr Plant Biol. 2026 Apr 2;68(9):3243–59. doi: 10.1111/jipb.70249 (PMC13545857; doi:10.1111/jipb.70249)
Supplement: Supplementary file 1 [file JIPB-68-3243-s001.docx]

**Enhancing CRISPR-Cas12a base editing in plants with LbCas12a variants and introns**

Yanhao Cheng^1,†^, Gen Li^1, †^, Man Zhou^1^, Rushil Mandlik^1^, Doris Wang^1^, Yiping Qi^1,2^

^1^Department of Plant Science and Landscape Architecture, University of Maryland, College Park, MD 20742, USA.

^2^Institute for Bioscience and Biotechnology Research, University of Maryland, Rockville, Maryland 20850, USA.

^†^These authors contributed equally to this work.

*Correspondence: Yiping Qi ([yiping@umd.edu](mailto:yiping@umd.edu))

**SUPPORTING INFORMATION**

**Table S1.** Oligos used in this study.

**Table S2.** crRNAs used in this study.

**Figure S1.** Co-editing outcomes of multiplex base editing mediated by dLbCas12a variant–based cytosine base editors in T_0_ rice stable lines.

**Figure S2.** Co-editing outcomes of multiplex base editing mediated by dLbCas12a variant–based adenine base editors in T_0_ rice stable lines.

**Figure S3.** Zygosity classification of T_0_ rice plants edited by dLbCas12a variant–based adenine base editors.

**Figure S4.** The A-to-G editing window analysis of four dLbCas12a variant-based A-to-G editors in rice T_0_ lines.

**Figure S5.** Genotypes of T_0_ rice plants edited by dLbCas12a-RRV-intron-CBE for whole-genome sequencing (WGS).

**Figure S6.** Genotypes of T_0_ rice plants edited by dLbCas12a-RRV-intron-ABE for whole-genome sequencing (WGS).

| **SUPPLEMENTAL TABLES**  **Table S1. Oligos used in this study** | | |
| --- | --- | --- |
| **Name** | **Sequence 5'-3'** | **Description** |
| Cpf1-OsROC5-gR1-F | TAGATTGCTTCCTGCAATGCCGGTAGAC | Forward primer for generating target A5 |
| Cpf1-OsROC5-gR1-R | GGCCGTCTACCGGCATTGCAGGAAGCAA | Reverse primer for generating target A5 |
| AC-crRNA2-F | TAGATGTCTCCCTGCAAACCACCGCGCA | Forward primer for generating target AC02 |
| AC-crRNA2-R | GGCCTGCGCGGTGGTTTGCAGGGAGACa | Forward primer for generating target AC02 |
| OsPDS-FokI-crRNA05-F | TAGATTTCAAAACCCTTAGAGATATCTA | Forward primer for generating target D5 |
| OsPDS-FokI-crRNA05-R | GGCCTAGATATCTCTAAGGGTTTTGAAA | Forward primer for generating target D5 |
| TTTN-OsmiR528-crRNA2-F | TAGATcctctctctCCTgtgcttgcctc | Forward primer for generating target L8 |
| TTTN-OsmiR528-crRNA2-R | GGCCgaggcaagcacAGGagagagaggA | Forward primer for generating target L8 |
| OsDEP1-cR2-23nt-F | tagatCAGAAAGAGAAGGAGGCACAGAT | Forward primer for generating target A4 |
| OsDEP1-cR2-23nt-R | ggccATCTGTGCCTCCTTCTCTTTCTGa | Forward primer for generating target A4 |
| CG-crRNA2-F | TAGATGCACCATATGCTTGCTGATCAAA | Forward primer for generating target CG02 |
| CG-crRNA2-R | GGCCTTTGATCAGCAAGCATATGGTGCa | Forward primer for generating target CG02 |
| AA-crRNA2-F | TAGATCCGGTGAAAAGGACCTTGTCCCA | Forward primer for generating target AA02 |
| AA-crRNA2-R | GGCCTGGGACAAGGTCCTTTTCACCGGa | Forward primer for generating target AA02 |
| CA-crRNA1-F | TAGATTACGTGGAAACAATGACAGTTCA | Forward primer for generating target CA01 |
| CA-crRNA1-R | GGCCTGAACTGTCATTGTTTCCACGTAa | Forward primer for generating target CA01 |
| HiTom-A5-F2 | gagttggatgctggatggTCTGACATGTTCTCGTGCAT | HiTom primer for target A5 |
| Hi-TOM-A5-R2 | ggagtgagtacggtgtgcCACTGTTGATCAGCACTCAC | HiTom primer for target A5 |
| HiTom-AC02-TTTV-F2 | ggagtgagtacggtgtgcTGCTCCCTTGGGGTGTTCGT | HiTom primer for target AC02-TTTV |
| HiTom-AC02-TTTV-R2 | gagttggatgctggatggCCTCTGCAGCCTGGTAAGCA | HiTom primer for target AC02-TTTV |
| HiTom-D5-F | gagttggatgctggatggGGAGATTGGTATGAAACTGGGC | Hi-TOM primer for target TTTTG05 |
| HiTom-D5-R | ggagtgagtacggtgtgcCCATGTGTGTTAATCCTCAGAC | Hi-TOM primer for target TTTTG05 |
| Hi-TOM-L8-F | ggagtgagtacggtgtgcTTTGGTTTGGGATAGGTAGGTG | HiTom primer for target TTTTG08 |
| Hi-TOM-L8-R | gagttggatgctggatggTTTCTCCTACCGCTGCTGAT | HiTom primer for target TTTTG08 |
| HiTom-A4-F | ggagtgagtacggtgtgcCATTTTAAGCATGTTCGCTG | HiTom primer for target TTTTC04 |
| HiTom-A4-R | gagttggatgctggatggGATCAAAACATACCCGATCC | HiTom primer for target TTTTC04 |
| Hi-TOM-AA2-F | ggagtgagtacggtgtgcCCCTGAAAAACAAAATGACCA | HiTom primer for target AA02-TTTV |
| Hi-TOM-AA2-R | gagttggatgctggatggCGAGTTTGCACCGTCAAC | HiTom primer for target AA02-TTTV |
| Hi-TOM-CA1-F | ggagtgagtacggtgtgcAGAGGAGAGCTGCATCCTGA | HiTom primer for target CA01-TTTV |
| Hi-TOM-CA1-R | gagttggatgctggatggCAGAATGGAATTTTGATTAAAAAGG | HiTom primer for target CA01-TTTV |
| Hi-TOM-CG2-TTTV-F | ggagtgagtacggtgtgcAAGACAGATTCCTCTGCATTGA | HiTom primer for target CG02-TTTV |
| Hi-TOM-CG2-TTTV-R | gagttggatgctggatggGCGTCAAACTTTCCTTAACAGG | HiTom primer for target CG02-TTTV |
| HiTom-CG02-TTN-F | ggagtgagtacggtgtgcTTACAGTGGTGCAGTCTGT | HiTom primer for target CG02-TTN |
| HiTom-CG02-TTN-R | gagttggatgctggatggGAATACAAAGAAATAACAAAATGCAAG | HiTom primer for target CG02-TTN |
| Hi-TOM-AA2-TTN-F | ggagtgagtacggtgtgcGCCACCGCTAAGTTCCTCG | HiTom primer for target AA02-TTV |
| Hi-TOM-AA2-TTN-R | gagttggatgctggatggGTCGAAGATGCAGGGGCAC | HiTom primer for target AA02-TTV |
| Hi-TOM-CA1-TTN-F | ggagtgagtacggtgtgcGAAAGCAGAGGAGAGCTGCG | HiTom primer for target CA01-TTV |
| Hi-TOM-CA1-TTN-R | gagttggatgctggatggGAAGGGACTGATTAGTGCCAAA | HiTom primer for target CA01-TTV |
| Hi-TOM-F-1-correct | ACACTCTTTCCCTACACGACGCTCTTCCGATCTgcttGCGTtggagtgagtacggtgtgc | Hi-TOM barcoding primer |
| Hi-TOM-F-2-correct | ACACTCTTTCCCTACACGACGCTCTTCCGATCTgcttGTAGtggagtgagtacggtgtgc | Hi-TOM ba rcoding primer |
| Hi-TOM-F-3-correct | ACACTCTTTCCCTACACGACGCTCTTCCGATCTgcttACGCtggagtgagtacggtgtgc | Hi-TOM barcoding primer |
| Hi-TOM-F-4-correct | ACACTCTTTCCCTACACGACGCTCTTCCGATCTgcttCTCGtggagtgagtacggtgtgc | Hi-TOM barcoding primer |
| Hi-TOM-F-5-correct | ACACTCTTTCCCTACACGACGCTCTTCCGATCTgcttGCTCtggagtgagtacggtgtgc | Hi-TOM barcoding primer |
| Hi-TOM-F-6-correct | ACACTCTTTCCCTACACGACGCTCTTCCGATCTgcttAGTCtggagtgagtacggtgtgc | Hi-TOM barcoding primer |
| Hi-TOM-F-7-correct | ACACTCTTTCCCTACACGACGCTCTTCCGATCTgcttCGACtggagtgagtacggtgtgc | Hi-TOM barcoding primer |
| Hi-TOM-F-8-correct | ACACTCTTTCCCTACACGACGCTCTTCCGATCTgcttGATGtggagtgagtacggtgtgc | Hi-TOM barcoding primer |
| Hi-TOM-F-9-correct | ACACTCTTTCCCTACACGACGCTCTTCCGATCTgcttATACtggagtgagtacggtgtgc | Hi-TOM barcoding primer |
| Hi-TOM-F-10-correct | ACACTCTTTCCCTACACGACGCTCTTCCGATCTgcttCACAtggagtgagtacggtgtgc | Hi-TOM barcoding primer |
| Hi-TOM-F-11-correct | ACACTCTTTCCCTACACGACGCTCTTCCGATCTgcttGTGCtggagtgagtacggtgtgc | Hi-TOM barcoding primer |
| Hi-TOM-F-12-correct | ACACTCTTTCCCTACACGACGCTCTTCCGATCTgcttACTAtggagtgagtacggtgtgc | Hi-TOM barcoding primer |
| Hi-TOM-R-A | GACTGGAGTTCAGACGTGTGCTCTTCCGATCTctgtGCGTtgagttggatgctggatgg | Hi-TOM barcoding primer |
| Hi-TOM-R-B | GACTGGAGTTCAGACGTGTGCTCTTCCGATCTctgtGTAGtgagttggatgctggatgg | Hi-TOM barcoding primer |
| Hi-TOM-R-C | GACTGGAGTTCAGACGTGTGCTCTTCCGATCTctgtACGCtgagttggatgctggatgg | Hi-TOM barcoding primer |
| Hi-TOM-R-D | GACTGGAGTTCAGACGTGTGCTCTTCCGATCTctgtCTCGtgagttggatgctggatgg | Hi-TOM barcoding primer |
| Hi-TOM-R-E | GACTGGAGTTCAGACGTGTGCTCTTCCGATCTctgtGCTCtgagttggatgctggatgg | Hi-TOM barcoding primer |
| Hi-TOM-R-F | GACTGGAGTTCAGACGTGTGCTCTTCCGATCTctgtAGTCtgagttggatgctggatgg | Hi-TOM barcoding primer |
| Hi-TOM-R-G | GACTGGAGTTCAGACGTGTGCTCTTCCGATCTctgtCGACtgagttggatgctggatgg | Hi-TOM barcoding primer |
| Hi-TOM-R-H | GACTGGAGTTCAGACGTGTGCTCTTCCGATCTctgtGATGtgagttggatgctggatgg | Hi-TOM barcoding primer |
| 4CL1-crRNA1-F | TAGATGCAACCCTGTAGTCCCTGATGAA | Forward primer for generating target 4CL1-crRNA1 in poplar |
| 4CL1-crRNA1-R | GGCCTTCATCAGGGACTACAGGGTTGCA | Reverse primer for generating target 4CL1-crRNA1 in poplar |
| 4CL1-crRNA2-F | TAGATCCAAGGAACCATTCGACATAAAA | Forward primer for generating target 4CL1-crRNA2 in poplar |
| 4CL1-crRNA2-R | GGCCTTTTATGTCGAATGGTTCCTTGGA | Reverse primer for generating target 4CL1-crRNA2 in poplar |
| Pll-crRNA1-F | TAGATAAACCTCTCAAGTTGAAGAATTC | Forward primer for generating target Pll-crRNA1 in poplar |
| Pll-crRNA1-R | GGCCGAATTCTTCAACTTGAGAGGTTTA | Reverse primer for generating target Pll-crRNA1 in poplar |
| Pll-crRNA2-F | TAGATAACCACCTTGAGCCCCAAAGCCT | Forward primer for generating target Pll-crRNA2 in poplar |
| Pll-crRNA2-R | GGCCAGGCTTTGGGGCTCAAGGTGGTTA | Reverse primer for generating target Pll-crRNA2 in poplar |
| SVP-crRNA1-F | TAGATCACTCGAAGAATCTTGAGAAGCT | Forward primer for generating target SVP-crRNA1 in poplar |
| SVP-crRNA1-R | GGCCAGCTTCTCAAGATTCTTCGAGTGA | Reverse primer for generating target SVP-crRNA1 in poplar |
| SVP-crRNA2-F | TAGATTCCGCAACTTCCTTACTCAACCT | Forward primer for generating target SVP-crRNA2 in poplar |
| SVP-crRNA2-R | GGCCAGGTTGAGTAAGGAAGTTGCGGAA | Reverse primer for generating target SVP-crRNA2 in poplar |
| 4CL1-crRNA3-F | tagatCTCTCGAGGCCTTGGCATGTTTT | forward primer for generating target 4CL1-crRNA3 in poplar |
| 4CL1-crRNA3-R | ggccAAAACATGCCAAGGCCTCGAGAGa | reversed primer for generating target 4CL1-crRNA3 in poplar |
| 4CL1-crRNA4-F | tagatTTAATAGCCCATCCAGAGATATC | forward primer for generating target 4CL1-crRNA4 in poplar |
| 4CL1-crRNA4-R | ggccGATATCTCTGGATGGGCTATTAAa | reversed primer for generating target 4CL1-crRNA4 in poplar |
| PII-crRNA3-F | tagatTAAACCATACCTCTTGCTTTAAC | forward primer for generating target PII-crRNA3 in poplar |
| PII-crRNA3-R | ggccGTTAAAGCAAGAGGTATGGTTTAa | reversed primer for generating target PII-crRNA3 in poplar |
| PII-crRNA4-F | tagatCGAGACTCGCCAGGGCCTATTTC | forward primer for generating target PII-crRNA4 in poplar |
| PII-crRNA4-R | ggccGAAATAGGCCCTGGCGAGTCTCGa | reversed primer for generating target PII-crRNA4 in poplar |
| SVP-crRNA3-F | tagatTTGAAAAGCCCTCTTCTTCGTTT | forward primer for generating target SVP-crRNA3 in poplar |
| SVP-crRNA3-R | ggccAAACGAAGAAGAGGGCTTTTCAAa | reversed primer for generating target SVP-crRNA3 in poplar |
| SVP-crRNA4-F | tagatCTCCAGCTTCTCAAGATTCTTCG | forward primer for generating target SVP-crRNA4 in poplar |
| SVP-crRNA4-R | ggccCGAAGAATCTTGAGAAGCTGGAGa | reversed primer for generating target SVP-crRNA4 in poplar |
| 4CL1-Hitom-F3 | ggagtgagtacggtgtgcGGGTGCTTGCACTTTTCAGA | HiTom primers for target 4CL1-crRNA1 in poplar |
| 4CL1-Hitom-R3 | gagttggatgctggatggCTCCATCTACCTGTTGAGCCA | HiTom primers for target 4CL1-crRNA1 in poplar |
| 4CL1-Hitom-F4 | ggagtgagtacggtgtgcGGGATATGGAATGACCGAGG | HiTom primers for target 4CL1-crRNA2 in poplar |
| 4CL1-Hitom-R4 | gagttggatgctggatggTCATGATCTGATCACCCCGG | HiTom primers for target 4CL1-crRNA2 in poplar |
| PII-Hitom-F1 | ggagtgagtacggtgtgcAAACCAGGCTCCCTCACTTC | HiTom primers for target PII-crRNA1 in poplar |
| PII-Hitom-R1 | gagttggatgctggatggCGCATACCAGGAACAGAGGA | HiTom primers for target PII-crRNA1 in poplar |
| PII-Hitom-F2 | ggagtgagtacggtgtgcGGCTCTGTTGAAAATTGGTATTCG | HiTom primers for target PII-crRNA2 in poplar |
| PII-Hitom-R2 | gagttggatgctggatggGCGAAAGGAGACGAAACAGAA | HiTom primers for target PII-crRNA2 in poplar |
| SVP-Hitom-F1 | ggagtgagtacggtgtgcCTTCCAGGCCATCTTAGGAAATGA | HiTom primers for target SVP-crRNA1 in poplar |
| SVP-Hitom-R1 | gagttggatgctggatggGCACTGTCTTAAACACTCTCCAC | HiTom primers for target SVP-crRNA1 in poplar |
| SVP-Hitom-F2 | ggagtgagtacggtgtgcCCTGACTTCTTTTACATTAGCTGTGG | HiTom primers for target SVP-crRNA2 in poplar |
| SVP-Hitom-R2 | gagttggatgctggatggAGGCATAGAGCTGCTAGAAGC | HiTom primers for target SVP-crRNA2 in poplar |
| 4CL1-Hitom-F5 | ggagtgagtacggtgtgcTTCCTACCAAGTTCACCTGA | HiTom primers for target 4CL1-crRNA3 in polpar |
| 4CL1-Hitom-R5 | gagttggatgctggatggAGTCCACGCACATGACCTTA | HiTom primers for target 4CL1-crRNA3 in polpar |
| 4CL1-Hitom-F6 | ggagtgagtacggtgtgcCGATATCGGCTACATTGATG | HiTom primers for target 4CL1-crRNA4 in polpar |
| 4CL1-Hitom-R6 | gagttggatgctggatggCCGCATCCTCATCTTTCAAT | HiTom primers for target 4CL1-crRNA4 in polpar |
| PII-HiTom-F3 | ggagtgagtacggtgtgcGTTCTATCCAATGTTGGCCC | HiTom primers for target PII-crRNA3 in polpar |
| PII-HiTom-R3 | gagttggatgctggatggCTTAGGCTAGTGGTATCATG | HiTom primers for target PII-crRNA3 in polpar |
| PII-HiTom-F4 | ggagtgagtacggtgtgcGGTAATCTTCTTCTTCGGCA | HiTom primers for target PII-crRNA4 in polpar |
| PII-HiTom-R4 | gagttggatgctggatggGGCAGCGAACTTTTAGAAGT | HiTom primers for target PII-crRNA4 in polpar |
| SVP-Hitom-F3 | ggagtgagtacggtgtgcTATTCTCTGTGGCTACCCTT | HiTom primers for target SVP-crRNA3 in polpar |
| SVP-Hitom-R3 | gagttggatgctggatggAAGACCTTGAGCTGGAGAAC | HiTom primers for target SVP-crRNA3 in polpar |
| SVP-Hitom-F4 | ggagtgagtacggtgtgcACTTCCAGGCCATCTTAGGA | HiTom primers for target SVP-crRNA4 in polpar |
| SVP-Hitom-R4 | gagttggatgctggatggACATACTCTATACATCATCCCAT | HiTom primers for target SVP-crRNA4 in polpar |
| OsACCase-crRNA01-TTV-F | tagatACCAGCCTATTATTCTTACAGGC | crRNA for Cas12a editing |
| OsACCase-crRNA01-TTV-R | ggccGCCTGTAAGAATAATAGGCTGGTa | crRNA for Cas12a editing |
| OsACCase-crRNA02-TTV-F | tagatGTGTGGAGAATATACATGGAAGT | crRNA for Cas12a editing |
| OsACCase-crRNA02-TTV-R | ggccACTTCCATGTATATTCTCCACACa | crRNA for Cas12a editing |
| OsACCase-crRNA03-TTTV-F | tagatCAGATTCTGCAACCAAGACTGCG | crRNA for Cas12a editing |
| OsACCase-crRNA03-TTTV-R | ggccCGCAGTCTTGGTTGCAGAATCTGa | crRNA for Cas12a editing |
| OsACCase-HiTom-F1 | ggagtgagtacggtgtgcGGCACTTCTGTCATAGCACA | HiTom primers for genotyping of LbCas12a-RRV based BEs |
| OsACCase-HiTom-R1 | gagttggatgctggatggCGCCTTCAAGGTCATCTGAA | HiTom primers for genotyping of LbCas12a-RRV based BEs |
| OsACCase-HiTom-F2 | ggagtgagtacggtgtgcTACTGGCAGAGCAAAGCTTG | HiTom primers for genotyping of LbCas12a-RRV based BEs |
| OsACCase-HiTom-R2 | gagttggatgctggatggGCAGGCTGATTGTATGTCCT | HiTom primers for genotyping of LbCas12a-RRV based BEs |
| OsACCase-sanger-F | GTTACCAACCTAGCCTGT | sanger sequencing primer for genotyping OsACCase- crRNA02 site |
| OsACCase-sanger-R | TGTAGGCAGGAACATAACT | sanger sequencing primer for genotyping OsACCase- crRNA02 site |

| **Table S2. crRNAs used in this study** | | | |
| --- | --- | --- | --- |
| **Targeted gene** | **Targeted site** | **PAM** | **Spacer sequence with PAM (5’- 3’)** |
| LOC_Os05g22940 | OsACCase-crRNA01 | CTTG | ACCAGCCTATTATTCTTACAGGC |
| LOC_Os05g22940 | OsACCase-crRNA02 | CTTG | GTGTGGAGAATATACATGGAAGT |
| LOC_Os05g22940 | OsACCase-crRNA03 | TTTC | CAGATTCTGCAACCAAGACTGCG |
| LOC_Os02g45250 | A5 | TTTC | TGCTTCCTGCAATGCCGGTAGAC |
| LOC_Os03g48170 | AC02 | TTTC | GTCTCCCTGCAAACCACCGCGCA |
| LOC_Os03g08570 | D5 | TTTG | TTCAAAACCCTTAGAGATATCTA |
| Osa-miR528 | L8 | TTTG | CCTCTCTCTCCTGTGCTTGCCTC |
| LOC_Os09g26999 | A4 | TTTC | CAGAAAGAGAAGGAGGCACAGAT |
| LOC_Os07g40404 | CG02-TTTV | TTTG | GCACCATATGCTTGCTGATCAAA |
| LOC_Os02g43194 | AA02-TTTV | TTTA | CCGGTGAAAAGGACCTTGTCCCA |
| LOC_Os07g10860 | CA01-TTTV | TTTA | TACGTGGAAACAATGACAGTTCA |
| LOC_Os07g40790 | CG02-TTV | CTTG | GCACCATATGCTTGCTGATCAAA |
| LOC_Os04g45720 | AA02-TTV | ATTA | CCGGTGAAAAGGACCTTGTCCCA |
| LOC_Os07g47284 | CA01-TTV | CTTA | TACGTGGAAACAATGACAGTTCA |
| PtXaAlbH.01G031600  PtXaTreH.01G031400 | 4CL1-crRNA1 | TTTG | GCAACCCTGTAGTCCCTGATGAA |
|  | 4CL1-crRNA2 | TTTG | CCAAGGAACCATTCGACATAAAA |
| PtXaAlbH.02G161100  PtXaTreH.02G161500 | PII-crRNA1 | TTTG | AAACCTCTCAAGTTGAAGAATTC |
|  | PII-crRNA2 | TTTG | AACCACCTTGAGCCCCAAAGCCT |
| PtXaAlbH.07G009900  PtXaTreH.07G009500 | SVP-crRNA1 | TTTG | CACTCGAAGAATCTTGAGAAGCT |
|  | SVP-crRNA2 | TTTC | TCCGCAACTTCCTTACTCAACCT |
| PtXaAlbH.01G031600  PtXaTreH.01G031400 | 4CL1-crRNA3 | TTTG | CTCTCGAGGCCTTGGCATGTTTT |
|  | 4CL1-crRNA4 | TTTG | TTAATAGCCCATCCAGAGATATC |
| PtXaAlbH.02G161100  PtXaTreH.02G161500 | PII-crRNA3 | TTTA | TAAACCATACCTCTTGCTTTAAC |
|  | PII-crRNA4 | CTTG | CGAGACTCGCCAGGGCCTATTTC |
| PtXaAlbH.07G009900  PtXaTreH.07G009500 | SVP-crRNA3 | TTTC | TTGAAAAGCCCTCTTCTTCGTTT |
|  | SVP-crRNA4 | GTTG | CTCCAGCTTCTCAAGATTCTTCG |

**SUPPLEMENTAL FIGURES**

**Figure S1. Co-editing outcomes of multiplex base editing mediated by dLbCas12a variant–based cytosine base editors in T_0_ rice stable lines.**

The number of co-edited target sites refers to the count of distinct target loci edited simultaneously within a single stable line. The co-editing rate was defined as the proportion of rice lines harboring n edited target sites relative to the total number of tested lines. Rice lines exhibiting C-to-T conversion frequencies greater than 5% at a given target site were classified as edited.

**Figure S2. Co-editing outcomes of multiplex base editing mediated by dLbCas12a variant–based adenine base editors in T_0_ rice stable lines.**

The number of co-edited target sites refers to the count of distinct target loci edited simultaneously within a single stable line. The co-editing rate was defined as the proportion of rice lines harboring n edited target sites relative to the total number of tested lines. Rice lines exhibiting A-to-G conversion frequencies greater than 5% at a given target site were classified as edited.

**Figure S3. Zygosity classification of T_0_ rice plants edited by dLbCas12a variant–based adenine base editors.**

Summary of A-to-G editing frequencies at seven endogenous target sites in rice T₀ plants edited by the indicated dLbCas12a variants-derived ABEs. T_0_ plants were categorized based on their A-to-G editing frequencies as follows: < 5%, wild-type (WT); 5%–30%, chimeric edit; 30%–75%, monoallelic edit; > 75%, biallelic edit. Mutation frequencies were determined by NGS of PCR amplicons, and all sequencing data were analyzed using CRISPR RGEN tools.

**Figure S4. The A-to-G editing window analysis of four dLbCas12a** **variant- based adenine base editors in rice T_0_ lines.**

**(A)** Editing window at target sites with TTTV PAMs. **(B)** Editing window at target sites with VTTV PAMs. Data are presented as mean values ± *SD* of 21–33 independent T_0_ lines.

**A**

dLbCas12a-RRV-

intron-CBE

Line #3

**B**

dLbCas12a-RRV-

intron-CBE

Line #5

**C**

dLbCas12a-RRV-

intron-CBE

Line #18

**Figure S5. Genotypes of T_0_ rice plants edited by dLbCas12a-RRV-intron-CBE for whole-genome sequencing (WGS).**

**(A**–**C)** Multiplex C-to-T base editing outcomes at four endogenous target sites in three independently edited rice T₀ plants: line # 3 **(A)**, line # 5 **(B)**, and line # 18 **(C)**. Cytosines within the protospacer are labeled as C1, C2, C3, etc., and highlighted in green. PAM sequences are highlighted in yellow. Numbers represent the percentage of thymine (T) among total sequencing reads at each cytosine position, as determined by NGS analysis of PCR amplicons.

**A**

dLbCas12a-RRV-intron-ABE

Line #2

**B**

dLbCas12a-RRV-intron-ABE

Line #20

dLbCas12a-RRV-intron-ABE

Line #27

**C**

**Figure S6. Genotypes of T_0_ rice plants edited by dLbCas12a-RRV-intron-ABE for whole-genome sequencing (WGS).**

**(A**–**C)** Multiplex A-to-G base editing outcomes at seven endogenous target sites in three independently edited rice T₀ plants: line # 2 **(A)**, line # 20 **(B)**, and line # 27 **(C)**. Adenines within the protospacer are labeled as A1, A2, A3, etc., and highlighted in dark pink. PAM sequences are highlighted in yellow. Numbers represent the percentage of guanine (G) among total sequencing reads at each adenine position, as determined by NGS analysis of PCR amplicons.
